# Investigation of factors affecting the efficacy of 3C23K, a human monoclonal antibody targeting MISIIR

**DOI:** 10.18632/oncotarget.19620

**Published:** 2017-07-27

**Authors:** Sarah E. Gill, Qing Zhang, Gary L. Keeney, William A. Cliby, S. John Weroha

**Affiliations:** ^1^ Department of Gynecologic Oncology, Mayo Clinic, Rochester, Minnesota, USA; ^2^ Department of Laboratory Medicine and Pathology, Mayo Clinic, Rochester, Minnesota, USA; ^3^ Department of Medical Oncology, Mayo Clinic, Rochester, Minnesota, USA

**Keywords:** 3C23K, mullerian inhibiting substance receptor, ovarian cancer, targeted therapy, patient-derived xenograft

## Abstract

MISIIR is a potential target for ovarian cancer (OC) therapy due to its tissue-specific pattern of expression. 3C23K is a novel therapeutic monoclonal anti-MISIIR antibody designed to recruit effector cells and promote cell death through ADCC (antibody dependent cell-mediated cytotoxicity). Our objective was to determine the tolerability and efficacy of 3C23K in OC patient-derived xenografts (PDX) and to identify factors affecting efficacy. Quantitative RT-PCR, immunohistochemistry (IHC), and flow cytometry were used to categorize MISIIR expression in established PDX models derived from primary OC patients. We selected two high expressing models and two low expressing models for *in vivo* testing. One xenograft model using an MISIIR over-expressing SKOV3ip cell line (Z3) was a positive control. The primary endpoint was change in tumor size. The secondary endpoint was final tumor mass. We observed no statistically significant differences between control and treated animals. The lack of response could be secondary to a number of variables including the lack of known biomarkers of response, the low membrane expression of MISIIR, and a limited ability of 3C23K to induce ADCC in PDX models. Further study is needed to determine the magnitude of ovarian cancer response to 3C23K and also if there is a threshold surface expression to predict response.

## INTRODUCTION

Ovarian cancer is the deadliest gynecologic cancer, ranking fifth overall in cancer deaths, with a morality-to-incidence ratio of 63.9% and an estimated 22,280 new cases in the United States this year [1]. The majority of women have widespread intra-abdominal disease at the time of diagnosis, and the five year survival rate for these women is only about 40% after receiving standard therapy [2, 3]. Currently, the standard first-line treatment for ovarian cancer consists of surgical cytoreduction and platinum-based chemotherapy. Although this approach has proven to be the most effective treatment to date, many ovarian cancers exhibit primary platinum resistance, and most patients develop secondary platinum resistance during the course of their disease. In this setting, there is a paucity of approved targeted therapies. Accordingly, effective novel therapies are needed to improve survival rates for patients diagnosed with ovarian cancer, especially in its advanced stages and in the setting of platinum resistance.

Tissue-selective cell surface receptors are putative targets for antibody-based cancer therapies and there is growing evidence that Müllerian inhibiting substance receptor type II (MISIIR) may be suitable for such an approach. MISIIR protein expression is low or absent in benign gynecologic and non-gynecologic tissues while 65-69% of ovarian cancers across multiple histologic subtypes express moderate to high levels [4, 5]. Moreover, expression is not limited to ovarian tumor masses since 56% of ovarian cancer ascites has measurable MISIIR in malignant cells [6]. Irrespective of the downstream effects of receptor activation, the pattern of expression makes MISIIR a potential immunotherapy target with high tissue specificity.

An anti-MISIIR monoclonal antibody, 12G4, was developed as a novel therapeutic option in ovarian cancer [7] and further development led to the humanized monoclonal IgG1 antibody 3C23K, which retains the epitope recognition of 12G4 but exhibits higher binding affinity for MISIIR [8]. Two primary mechanisms of cytotoxicity have been reported, antibody-dependent cellular phagocytosis (ADCP) and antibody-dependent cytotoxicity (ADCC). However, the relative importance of ADCP and ADCC is partially dependent on the host immune system. For instance, murine natural killer (NK) cells exhibit minimal activation by human IgG1, such as 3C23K, while murine macrophages account for most of the *in vivo* efficacy seen in an ovarian granulosa cell tumor (GCT) xenograft engineered to overexpress MISIIR [8]. Since ovarian GCTs are less common than epithelial ovarian cancer (EOC), anti-MISIIR therapy was further tested in NIH-OVCAR-3 cell line xenografts and demonstrated significant tumor growth inhibition [9]. Taking the other, preliminary studies indicate that 3C23K or 12G4 has activity in EOC and ovarian GCT cell lines with ADCP as the primary mechanism of immune mediated cytotoxicity. However, it is unclear if the responses seen in these cell lines are representative of primary EOC or if the endogenous expression of MISIIR is a sufficient marker to select tumors with the greatest likelihood of response to 3C23K or 12G4.

To test the efficacy of 3C23K in clinically relevant models of EOCs, patient-derived xenograft (PDX) models were utilized. In addition to recapitulating the histologic and molecular features of the source patient tumor, the *in vivo* response to chemotherapy is similar to that of the corresponding patient [10]. Although the animal host for these PDXs is the severe combined immunodeficient (SCID) beige mouse lacking functional B and T cells, these mice have retained intact complement [11], macrophage activity [12], and attenuated but active NK cells capable of lysing lymphoma YAC-19 cells [13, 14]. Secondary goals of this pilot study were to identify predictors and barriers of response, including receptor expression.

## RESULTS

### MISIIR expression in ovarian cancer PDX models

**Figure 1:**
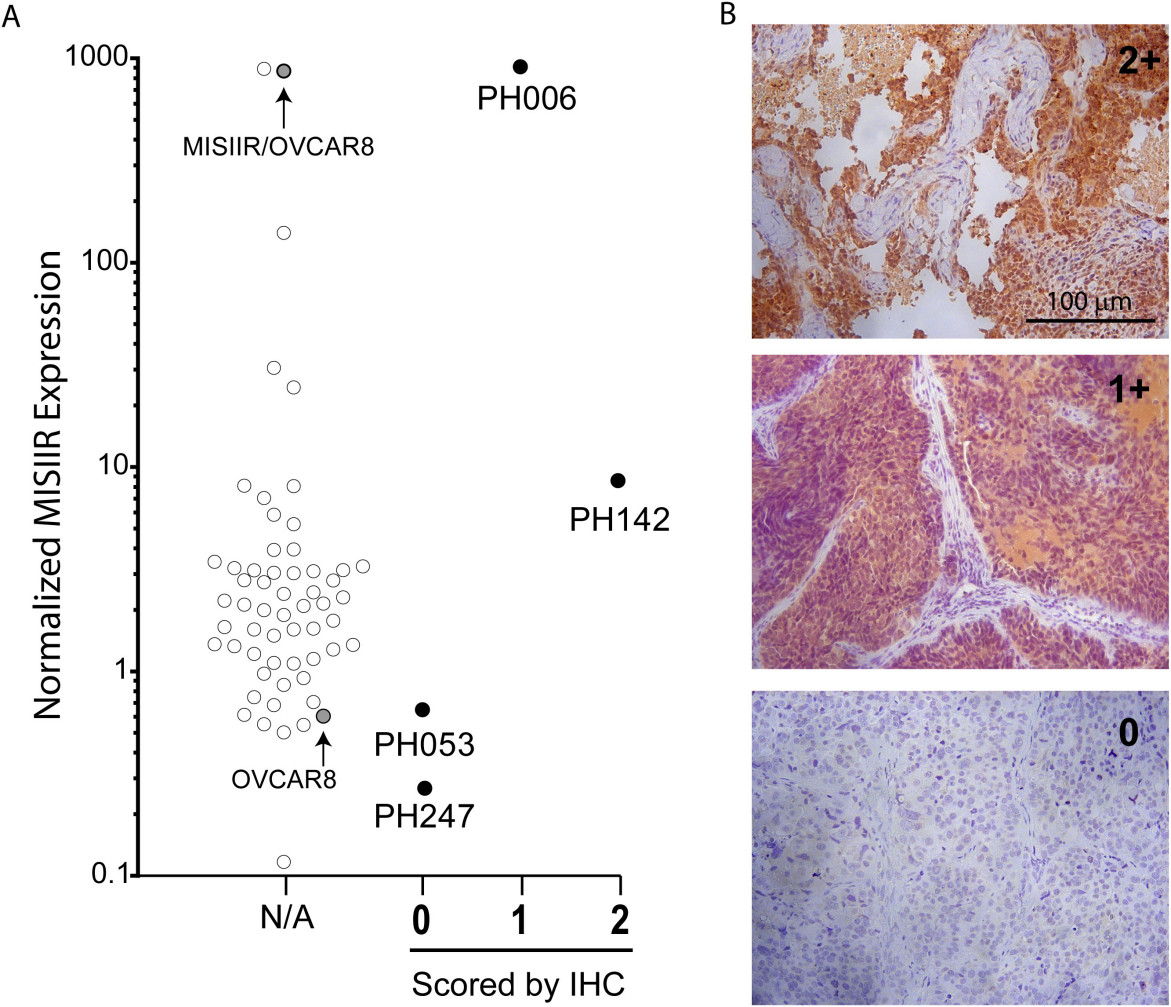
MISIIR mRNA and protein expression in selected PDX models **(A)** Composite of mRNA and protein expression levels of all OC models screened. MISIIR mRNA amplified by OriGene primers and normalized to housekeeping gene, RPL19. MISIIR/OVCAR8 (overexpressing cell line) and OVCAR8 (parental cell line) are included for comparison. IHC scores assigned by a gynecologic pathologist also represented, 2+ being strong staining and 0 being minimal to no staining, as described in Methods. **(B)** Representative images of PDX tumors at 200X magnification with IHC scores of 2+ (top), 1+ (middle), and 0 (bottom). These images represent staining seen in tumor tissues of PH142, PH006, and PH247 respectively.

Given the frequent expression of MISIIR in ovarian cancer [5] and the need for preclinical models to evaluate the efficacy of MISIIR targeting in ovarian cancer [15], this study evaluated the expression of MISIIR in the largest known resource of molecularly defined, histologically validated, and clinically annotated ovarian cancer PDX models [10]. Since 3C23K activity is presumed to be dependent on MISIIR expression, 75 individual PDX tumors were screened for mRNA expression by qRT-PCR and normalized to RPL19 (Figure 1A). The range of expression spanned three logs, 0.12 to 910 with the highest expression noted in an ovarian carcinosarcoma (PH006), surpassing the engineered MISRII+ cell line, MISIIR/OVCAR8. Although specific assessment of membrane bound MISIIR was considered using cellular fractionation techniques and Western blotting of MISIIR protein, the transforming growth factor beta (TGF-beta) superfamily of receptors is known to exhibit rapid membrane turnover [16]. Accordingly, measuring the expression of MISIIR at a static time point may not adequately reflect the cumulative dynamic expression of MISIIR available for binding by 3C23K over time.

MISIIR expression was also confirmed at the protein level as the 10 highest and lowest expressing models by mRNA were characterized by immunohistochemistry (IHC). Strong intensity of the cytoplasmic staining obscured the ability to specifically evaluate the intensity of membrane staining, so the intensity of both cytoplasmic and membrane staining was considered collectively (Figure 1B). Each PDX model was characterized as expressing a “high” (MISIIR-H) or “low” (MISIIR-L) level based on the level of MISIIR mRNA and protein expression. MISIIR-H models had mRNA expression exceeding a normalized ratio of 8.6 and an IHC score of 2 for protein expression. MISIIR-L models had normalized mRNA expression less than 0.65 and an IHC score of 0. Concordance of relative mRNA and protein expression was a requirement for inclusion in the efficacy phase of the study. Models PH006, PH142, PH053, and PH247 were ultimately selected. The clinical characteristics of the primary tumors used to establish these models are outlined in Table 1.

**Table 1:** Clinical and molecular characteristics of OC PDX primary tumor

OC PDX model	Histology	Stage	FIGO grade
PH006	carcinosarcoma/clear cell	3C	3
PH142	carcinosarcoma	1C	3
PH053	serous	3C	3
PH247	serous	3C	3

### MISIIR targeting *in vivo* with 3C23K

**Figure 2:**
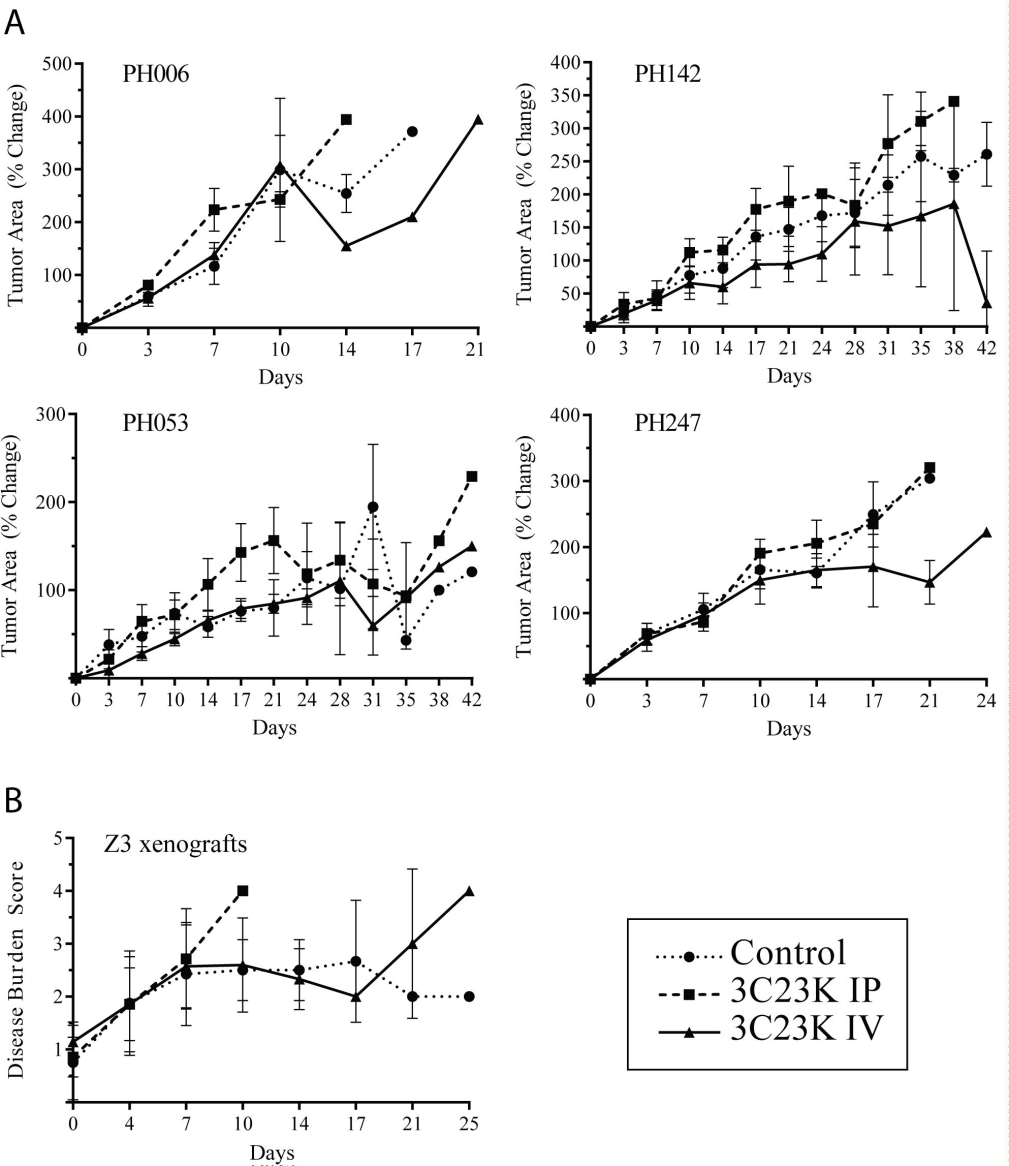
Efficacy of 3C23K in OC PDX models and Z3 xenografts **(A)** Percent change in tumor cross-sectional area over time in MISIIR-H (top) and MISIIR-L (bottom) OC PDX models as determined by ultrasound twice weekly. **(B)** Change in disease burden score in Z3 xenografts during efficacy studies. Values reported as percent change at each day of treatment as compared to the beginning of treatment, or day 0.

To maximize the therapeutic potential of 3C23K treatment, the dose administered in current studies was 10 and 5 times greater than previously reported efficacious doses of 12G4 [9] and 3C23K [8], respectively. In the absence of published data on safety and tolerability with 3C23K specifically in SCID beige mice, 10 animals were treated with 50 mg/kg intraperitoneal or intravenous injections twice weekly (n=5 each) as described in the Methods. There was no clinically significant weight loss or change in body conditioning scores [17] in any animals. After ensuring the tolerability of the study dose of 3C23K, MISIIR-H models (PH006 and PH142) and MISIIR-L models (PH053 and PH247) were engrafted, and when tumors reached 0.5 cm^2^, mice were randomized to receive normal saline, IP 3C23K, or IV 3C23K at the same dose and schedule. Across all four models, 94% of all mice (n = 120) did not reach the end of study (42 days post-initiation of treatment) due to progressive disease. The rate of progression was not different across any of the arms or between MISIIR-H and MISIIR-L PDX models. Within each model, the median survival was not statistically significantly different in any cohort across all PDX models: PH006: p=0.86, PH142: p=0.23, PH053: p=0.79, and PH247: p=0.87 (Table 2). The experimental arms receiving 3C23K in model PH053 appeared to differ from controls, but this result was driven primarily by 1-2 outliers in those arms (Table 2). Similarly, the change in tumor area from day 0 to animal sacrifice was not statistically significantly different between experimental and control arms in any of the PDX models (p range 0.16 to 0.88) (Figure 2A). Consistent with the ultrasound findings, we observed no significant differences in tumor mass at necropsy between control and IV or IP 3C23K-treated animals (ANOVA with Dunn’s multiple comparisons p > 0.05). For instance, the normalized tumor mass, defined as a ratio of the average tumor mass in treated animals relative to controls, was not significantly different from 1.0. These data indicate a lack of benefit from treatment (Table 2).

**Table 2:** Median survival and final tumor weight in PDX models and Z3 xenografts

Model ID	Treatment	Median survival in days (range)^a^	P value	Normalized final tumor weight (g) ± SEM^b^	P value
**PH006**	Normal Saline 3C23K IP 3C23K IV	8.5 (3, 17) 7 (7, 14) 8.5 (3, 21)	0.8569	1.02 ± 0.21 0.95 ± 0.08	0.7590
**PH142**	Normal Saline 3C23K IP 3C23K IV	35 (7, 42) 21 (10, 38) 29.5 (10, 42)	0.2287	0.96 ± 0.08 0.88 ± 0.14	0.6258
**PH053**	Normal Saline 3C23K IP 3C23K IV	10 (3, 42) 21 (7, 42) 24 (10, 42)	0.7907	1.40 ± 0.12 1.20 ± 0.12	0.2539
**PH247**	Normal Saline 3C23K IP 3C23K IV	10 (7, 21) 14 (10, 21) 14 (7, 24)	0.8668	0.96 ± 0.07 0.96 ± 0.04	1.000
**Z3 xenografts**	Normal Saline 3C23K IP 3C23K IV	12.5 (4, 29) 11 (7, 11) 12 (7, 29)	0.4605	1.62 ± 0.16 1.61 ± 0.22	0.9711

^a^Median survival is reported as the median number of days the mice survived after the first administration of 3C23K.

^b^Final tumor weight is normalized to the mean tumor weight of mice in the control group.

Given the lack of efficacy in SCID beige mice, additional studies explored whether response is dependent on the robustness of natural killer (NK) cell activity, which differs between strains of mice. For instance, athymic nude mice are known to maintain NK cell activity [18] while SCID beige mice exhibit decreased but intact NK cell activity [13]. To optimize experimental conditions, an engineered cell line (Z3) with confirmed membrane expression of MISIIR by flow cytometry and immunofluorescence was established intraperitoneally in athymic nude mice and treated using the same scheme as noted above. Survival was not statistically significantly different between arms (normal saline: 12.5 days, range 4-29 days; 3C23K IP: 11.0 days, range 7-11 days; 3C23K IV: 12.0 days, range 7-29 days; p=0.4605). There were also no differences in final tumor weight (Table 2) or disease burden (Figure 2B). Taken together, these data suggest that NK cell activity alone is not sufficient to confer sensitivity to MISIIR treatment.

### Assessment of membrane receptor expression in PDX models

**Figure 3:**
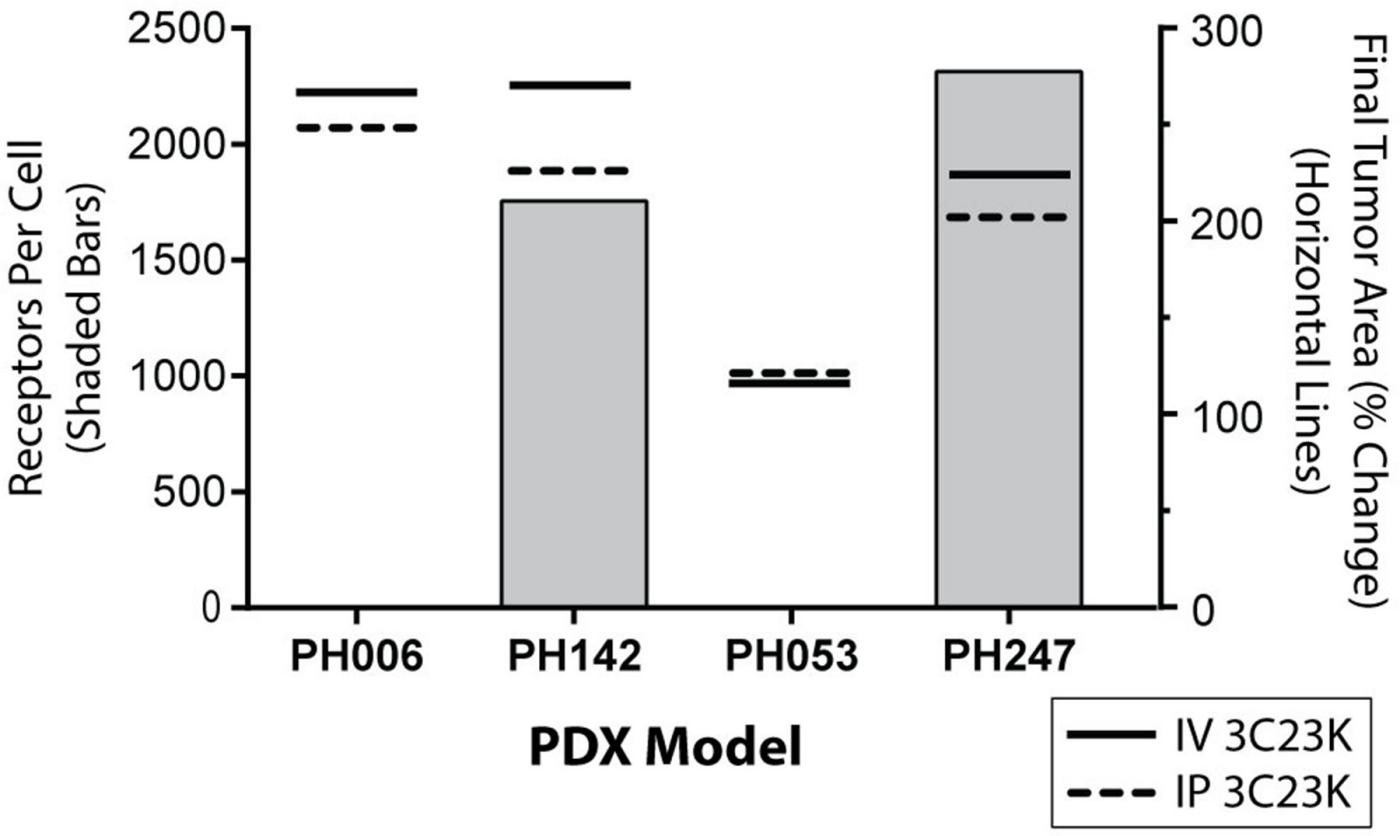
MISIIR membrane receptor density in OC PDX tumors compared with *in vivo* response The membrane density of MISIIR is expressed as number of receptors per cell (X axis). The final PDX percent change in tumor size from baseline is an average of all mice in the respective IV or IP cohorts (Y axis), regardless of when they were sacrificed.

An important goal of the study was to understand predictors of response to 3C23K which could serve as biomarkers to inform future studies and identify future candidates for therapy. The primary mechanisms of action of 3C23K are presumed to be ADCP and ADCC after antibody binding to MISIIR in the presence of specific immune cells [7, 8]. As such, a minimum threshold number of surface membrane receptors may be required to trigger effector cell binding and induce cytolysis and phagocytosis of the cancer cells. Thus, we evaluated the membrane MISIIR density on cancer cells from the PDX models. Fresh tumor samples from untreated controls were analyzed by quantitative flow cytometry. When compared to total cellular expression levels (mRNA and protein), the MISIIR-H models exhibited discordant membrane receptor density: PH006 (0) and PH142 (1755.5) (Figure 3). Similar results were observed with MISIIR-L models: PH053 (0), PH247 (2313.7). Taken together, mRNA or total protein expression levels are not predictive of surface density or response to 3C23K treatment.

## DISCUSSION

Novel and effective therapies are needed to improve survival rates for patients with advanced ovarian cancer. 3C23K is a novel immunotherapy designed to bind MISIIR on cancer cells and recruit effector cells, activating ADCP and ADCC by cytolysis and phagocytosis. Although the reported mechanisms of action of 3C23K portends great potential as a treatment for advanced ovarian cancer due to the selectivity and specificity of MISIIR expression, the specific requirements for *in vivo* response have not been defined yet. In our initial experience using PDX models, we did not observe a response to 3C23K. However, correlative studies offer several explanations for the lack of response, including low level of MISIIR surface expression in PDX models.

The lack of response to 3C23K cannot be explained by the immunodeficient mouse strain alone. For instance, macrophage activity has been shown to be a major contributor to immune mediated response with 3C23K [8] and this cell type is retained in SCID beige mice [12]. Indeed, when the relative impact of ADCP and ADCC were compared in nude mice treated with 3C23K, ADCP by murine macrophages more efficiently and consistently induced cell lysis than ADCC by murine peripheral blood mononuclear cells [8]. Although the relative contribution of ADCC by murine effector cells may be lower, it should be noted that the beige mutation in SCID mice does not eliminate the activity of NK cells. For instance, splenocytes (containing NK cells) are able to induce significant target lysis of YAC cells in a standard ^51^Cr release study [13]. Compared to beige wild type mice, the percent specific lysis was reduced by 50%, but not absent. In addition, SCID beige splenocytes readily lyse Be6 cells with efficiency comparable to wildtype splenocytes [13]. Nevertheless, 3C23K was tested in a nude mouse strain in order to study the efficacy in a more immunologically proficient animal host targeting Z3 xenograft cells overexpressing MISIIR.

The assumption that a certain threshold number of surface receptors is required to elicit a therapeutic response in cancer cells originates from the known mechanisms of action of 3C23K. Although the minimum number of surface receptors required to illicit a therapeutic response to 3C23K is unknown, previous studies have demonstrated *in vivo* response in a transfected granulosa cell tumor line (COV434) with 20,000 MISIIR receptors per cell and in NIH-OVCAR-3 cell lines, albeit to a lesser extent, with 4,000 receptors per cell [9]. Since the highest number of membrane receptors found in our PDX models was 2,300, it is possible but that the threshold density for efficacy was not reached in ovarian tumors that endogenously express MISIIR. Alternatively, since MISIIR expression has been shown to be fairly specific to gynecologic cancers and 3C23K is internalized by endocytosis [8], antibody-mediated delivery of toxic compounds (radioisotopes, catalytic toxins, drugs, cytokines and enzymes) could result in more significant reductions in tumor burden and prolongation of survival [19, 20].

Total mRNA and protein expression of MISIIR did not correlate with the number of receptors per cell. Our results suggest that quantification of mRNA expression and intensity of cytoplasmic IHC staining are not sufficient biomarkers of response. Although flow cytometry remains the standard for quantification of membrane proteins, it is not the ideal method for screening PDX tissue since fresh cells from mice are required and this approach would not be feasible on primary patient tumor specimens. Efficient methods to screen archived tissue would be more practical. For instance, formalin-fixed, paraffin-embedded tumor specimens are readily available from PDX tissue and archived patient clinical samples. However, to our knowledge, there is currently no validated method for detecting membrane bound MISIIR by IHC.

Other gynecologic cancers may be more responsive to 3C23K therapy. For instance, granulosa cell tumors frequently express MISIIR (96%-100% of samples studied) [21–24], and the expression is noted to be strongly membranous when overexpressed *in vitro* [9]. Similarly, other cancers may show higher levels of expression such as uterine sarcomas (owing to their Müllerian origin) [5]. Although studies report that 70%-80% of epithelial ovarian cancers express MISIIR [5, 23], the expression appears cytoplasmic [9] and on average is only detected in 50% of malignant cells.

Despite incremental improvements in overall survival over the last several decades [25], ovarian cancer remains the most lethal gynecologic cancer. Preclinical development of novel therapies like 3C23K is needed to help define the appropriate biomarkers for patient selection or stratification on future clinical trials. To this end, the recognition that most ovarian cancer cell lines are molecularly divergent from primary ovarian cancer [26] has helped fuel the interest in PDX models for early drug development [15, 27]. Indeed, the United States National Cancer Institute has transitioned away from drug screening *in vitro* while redirecting efforts to build a PDX repository in the developmental therapeutics program [28]. Although the data presented herein did not show efficacy in PDX models, these data are vital to the further development of 3C23K and may lead to more efficient discovery of positive data by limiting the duplication of effort.

## MATERIALS AND METHODS

### Antibodies and cell lines

Humanized anti-MISIIR monoclonal antibody, 3C23K, an IgG1 glyco-engineered by Emabling technology, was provided by GamaMabs Pharma (Toulouse, France). Monoclonal antibody 12G4 [9] was used for immunohistochemistry (IHC) and was provided by Isabelle Navarro-Teulon, Ph.D. (Montpellier, France). Established parent cell lines, OVCAR8 and SKOV3ip, were used to create the MISIIR transfected cell lines MISIIR/OVCAR8 and Z3 as previously described [5] and cells were obtained from American Type Culture Collection, (ATCC, Manassas, VA).

### MISIIR mRNA expression by quantitative RT-PCR

Measurement of mRNA expression was performed using a two-step real time polymerase chain reaction (qRT-PCR) process utilizing RNA extract from cryopulverized tumor samples of PDXs. The RNA was converted to cDNA using the High Capacity RNA-to-DNA kit (Applied Biosystems, Carlsbad, CA) on the BioRad iCycler (Hercules, CA). Gene-specific primers were used to amplify exons 7 and 8 of MISIIR (OriGene, Rockville, MD), forward primer: GCCTGGCATTTCTCCATGAGGA and reverse primer: CAGGTCTCCAATGGCACACGAT. The qRT-PCR protocol was performed on the LightCycler^®^ 480 II (Roche, Indianapolis, IN) as follows: denaturation at 95°C for 10 minutes followed by 40 cycles of 95°C for 15 seconds (s) and 60°C for 60 s with a final elongation step at 72°C for 60 s. The concentration of MISIIR mRNA was determined by fluorescence detection with Power SYBR green PCR Master mix (Applied Biosystems, Carlsbad, CA) in triplicate and normalized to the expression of the housekeeping gene, RPL19. Due to the relative abundance of RPL19, the normalized ratio of MISIIR:RPL19 was multiplied by a factor of 10^5^.

### MISIIR expression by immunohistochemistry

Tumor samples were obtained at the time of sacrifice from control mice in each model and fixed in formalin prior to embedding in paraffin. Tissue sections, 5-6 μm thick, were deparaffinized in serial xylene and rehydrated. Antigen retrieval was performed with citrate buffer pH 6.0 (Dako, Carpenteria, CA) in a 95-99°C water bath for 30 minutes followed by 5 minutes in H_2_O_2_ (Peroxidazed 1, BioCare Medical). Tissues were blocked with serum-free protein block (Dako, Carpenteria, CA) for 5 minutes, washed, then incubated with primary antibody, 12G4, overnight at 4°C in background reducing antibody diluent (Dako, Carpenteria, CA). Subsequent incubation with SignalStain Boost IHC detection reagent, horse radish peroxidase (HRP)-conjugated anti-mouse secondary antibody (Cell Signaling Technology, Danvers, MA,) was performed for one hour. Chromagen 3, 3’ diaminobenzidine (DAB) was used for visualization with a hematoxylin counterstain. Slides were de-identified and MISIIR expression scored by a pathologist specializing in gynecologic cancers, assigning scores on a scale of increasing intensity, with 0 representing minimal to no staining, 1 moderate staining, and 2 strong staining.

### Ovarian cancer xenografts and *in vivo* efficacy studies

To confirm the reported tolerability of 3C23K in athymic nude mouse strains (personal correspondence, GamaMabs Pharma), a short tolerability study was performed in 6-8 week old female severe combined immunodeficient (SCID) beige mice (ENVIGO/Harlan Laboratories, Indianapolis, IN), in accordance with Mayo Institutional Animal Care and Use Committee guidelines, as previously described [10]. The SCID mouse strain is preferred over athymic nude mice since human ovarian tumors exhibit poor engraftment in nude mice [29]. Tumor-bearing mice were used to account for the stress of having intraperitoneal disease while undergoing treatment. PDX tumors were revived from cryogenic storage and 0.1-0.3 cc of tumor fragments, without mechanical or enzymatic dissociation, were loaded into syringes with roughly equal volume of McCoy’s media for IP injection through a 16 gauge half-inch needle [10] in a total of 10 mice. Once tumor size was confirmed to be greater than 0.5 cm^2^ by ultrasound, 3C23K was administered at the dose determined by GamaMabs Pharma, 50 mg/kg twice weekly in normal saline IV (n= 5) and IP (n=5) for four weeks [30].

For efficacy studies, PDXs were established in the same fashion with IP injection of tumor cells and observed for 1-3 months for engraftment [10]. Although tumor palpation scoring has shown good correlation with caliper-based tumor measurements in these models [31], transabdominal ultrasound has been validated as reproducible to identify and quantitate tumor size during the pre-study and study period [10, 32]. Mice were randomized at a tumor threshold of approximately 0.5 cm^2^ in area by ultrasound into one of three experimental arms: (A) normal saline IP (n=5) or IV (n=5), (B) 3C23K 50 mg/kg IP (n=10) twice weekly or (C) 3C23K 50 mg/kg IV (n=10) twice weekly. Tumor growth was evaluated twice weekly by ultrasound measurement of the tumor area [10]. The primary endpoint was change in tumor area from baseline on day 42 or the date of sacrifice, calculated as the of tumor area in individual mice on day 42 or date of sacrifice divided by the corresponding tumor on day 1, and expressed as a group mean by cohort. Secondary endpoints were tumor mass and animal survival. Animals were sacrificed when the first of several specific criteria were met: end of study on day 42, tumor burden ≥10% of the mouse body weight, or body health score <6 [17]. For mice bearing ascites, the total tumor burden was defined as tumor weight plus weight of ascites at sacrifice.

To establish xenografts of engineered MISIIR overexpressing cell lines, MISIIR transfected SKOV3ip cells (Z3) (with expression confirmed by flow cytometry and immunofluorescence, data not shown) were injected intraperitoneally (1 x 10^6^ cells per ml) into athymic nude mice (ENVIGO/Harlan Laboratories). Tumors were allowed to grow to a size of 0.5 cm^2^ as measured by ultrasound or until the mice had obvious ascites by ultrasound. The Z3 xenografts were randomized, monitored, and treated exactly as the PDX models.

### Assessment of MISIIR membrane expression

Flow cytometry was utilized to analyze the membrane MISIIR density on cells from OC PDX tumors. Approximately 500 mg of fresh tumor was harvested, minced, and dissociated in a gentleMACS dissociator (Miltenyi Biotec Inc., San Diego, CA) following the standard manufacturer’s protocol with 5 ml of RPMI 1640 media and 1X with L-glutamine (Mediatech, Inc., Manassas VA). Briefly, the tumor was cut into 2-4 mm pieces and transferred into a gentleMACS tube containing RPMI and run on the programs h_tumor_01, h_tumor_02, and h_tumor_03 successively. The tumor-containing RPMI solution was then passed through a 70 μm mesh cell strainer to collect a single cell suspension. The cells were pelleted and resuspended in phosphate-buffered saline (PBS) at 1-2 x 10^6^ live cells per mL, viability assessed with trypan blue. MISIIR-transfected OVCAR8 cells incubated in 12G4 served as a positive control and tumor cells incubated in isotype control IgG1 antibody (Cell Signaling, Danvers, MA) served as a negative control. Quantitative flow cytometry analysis was carried out using Dako QIFIKIT (DakoCytomation, Copenhagen, Denmark) following the standard manufacturer’s protocol. Briefly, 1-2 x 10^6^ live cells were incubated for one hour at 4°C with escalating concentrations (from 5 μg/ml to 500 μg/ml) of the primary antibody, 12G4, or the isotype control IgG1 antibody in PBS containing 0.1% bovine serum albumin and 15 mmol/L sodium azide (NaN_3_). After washing with PBS/BSA/NaN_3_, cells were incubated for 45 minutes with FITC-conjugated anti-mouse IgG (DakoCytomation) at 4°C. The labeled samples were washed with the PBS/NaN_3_ and fixed in 1% paraformaldehyde overnight at 4°C. Cells were then washed and analyzed using a FACScan flow cytometer (FACSCanto, Becton Dickinson). Propidium iodide was used to exclude nonviable cells from analysis.

### Statistical analysis

Power analysis performed on JMP software (Cary, NC) demonstrated that 10 animals were required in each cohort for each PDX model chosen to show a 33% difference in final tumor area between arms, with a power of 80% and a standard deviation of 25%. A two-sided type I error rate of 0.05 was considered significant. The primary endpoint was change in tumor area; secondary endpoints were tumor weight and animal survival. Significant differences between the final tumor weights in experimental arms were determined using the Kruskal-Wallis test. Comparisons of tumor change from baseline by ultrasound between control and treated mice were performed by ANOVA with a Dunnett’s post hoc test for multiple comparisons. Survival was analyzed by Mantel-Cox log-rank tests. The membrane receptor density of MISIIR assessed by flow cytometry was analyzed by curvilinear regression using GraphPad Prism 6 software (GraphPad Software, La Jolla, CA).

## Abbreviations

ADCC: antibody dependent cell-mediated cytotoxicity
ATCC: American Type Culture Collection
C: Celsius
CD16: cluster of differentiation 16
cDNA: complement deoxyribonucleic acid
cm: centimeter
DAB: 3,3'-diaminobenzidine
EOC: epithelial ovarian cancer
Fc: fragment, crystallizable
FITC: fluorescein isothiocyanate
HRP: horse radish peroxidase
IgG: immunoglobulin G
IHC: immunohistochemistry
IP: intraperitoneal
IV: intravenous
kg: kilogram
mg: milligram
μg: microgram
MISIIR: Müllerian inhibiting substance receptor type 2
ml: milliliter
μl: microliter
mM: millimolar
mmol/L: millimoles per liter
mRNA: messenger ribonucleic acid
NaCl: sodium chloride
NaF: sodium fluoride
NaN_3_: sodium azide
NK: natural killer
nm: nanometer
PBS: phosphate-buffered saline
PDX: patient-derived xenograft
qRT-PCR: quantitative real time polymerase chain reaction
RPL19: ribosomal protein L19
RPMI: Roswell Park Memorial Institute medium
s: seconds
SCID: severe combined immunodeficient
